# Realising the broader value of vaccines in the UK

**DOI:** 10.1016/j.jvacx.2021.100096

**Published:** 2021-04-06

**Authors:** Simon Brassel, Margherita Neri, Phill O'Neill, Lotte Steuten

**Affiliations:** Office of Health Economics, London, United Kingdom

**Keywords:** Vaccination, Immunisation, Health Economics, Health Technology Assessment

## Abstract

Many health technology assessment (HTA) agencies limit their assessments of vaccines to the health benefits for the vaccinated individual, the costs associated with vaccine administration and the disease avoided. However, because the value of vaccines tends to accrue to a large extent beyond the vaccinated individual, they are systematically undervalued in many current HTA processes. This is also the case in the UK, where the Joint Committee on Vaccination and Immunisation (JCVI) is in charge of assessing preventative vaccines, while therapeutic vaccines fall in the realm of the National Institute for Clinical Excellence (NICE).

To contribute to a forward-looking perspective, we designed a framework to capture the broader value of vaccination. We reviewed the current state of the global vaccines pipeline and selected seven preventative and three therapeutic vaccines that are likely to enter the UK market within five years. We assessed on which value elements the selected vaccines would potentially generate value, and compared those against the novel broader value framework. A review of the current value elements considered by the JCVI and NICE allowed identifying the critical gaps between potential value generation and value recognition.

To our knowledge, this is the first time that the broader value of vaccination has been pro-actively assessed for pipeline vaccinations. Our findings show that the existing narrow evaluation frameworks are likely to systematically undervalue the value of potential future vaccines coming to the UK market. This is particularly relevant, where their impact on AMR and other health interventions, and on the productivity of the workforce is of concern. Recommendations to overcome this include an explicit and more consistent inclusion of, and data collection on, the impact of vaccines on AMR and other health interventions by JCVI and NICE; the consideration of a societal perspective and the fiscal impact of vaccines to societies.

## 1 Introduction

Vaccination programmes have been shown to be cost-effective or even cost-saving to the healthcare system and society [1], [2], [3], [4]. Moreover, the COVID-19 pandemic painfully exposes the human and economic costs of *not* having a vaccine available against highly infectious and potentially deadly disease. Central to the debate on how to respond to this pandemic are considerations of individual patient suffering, impact on patient’s families and loved ones, on people needing care for non-COVID-19 conditions, on healthcare workers and the broader healthcare system, and on the (global) economy affecting the population’s health and welfare today and in the future.

In this context, it may be surprising that many health technology assessment (HTA) bodies, organisations that conduct value assessment of vaccines to inform adoption and reimbursement decisions by policymakers, limit their assessments to the health benefits for the vaccinated individual and the costs associated with vaccination and the disease avoided [5]. Some will also consider herd-immunity [6] if data is available and when analytic expertise allows. The recent recommendation in some countries (e.g. the UK, the Netherlands) to vaccinate boys against HPV is an example and, so far, an exception.

Because the value of vaccines tends to accrue to a relatively large extent beyond the vaccinated individual, they are systematically undervalued in many current HTA processes [7]. Undervaluation can lead to underutilisation and reduces incentives for manufacturers to innovate. Health economists have therefore long called for explicitly considering the ‘broader value aspects’ of vaccines, including for example cost offsets to the broader healthcare system and society [8], [9], or potential impact on antimicrobial resistance (AMR) by preventing diseases that otherwise would require antibiotic treatment [10].

Arguments provided by HTA bodies for using a more narrow perspective, in practice if not on paper, are that the perspective adopted for economic evaluations should be the same for all interventions, that sufficient data are lacking [7], that analytic ability to incorporate such impact is suboptimal [9], [11] and/or that the understanding of relevant decision-making processes is insufficient to change the status quo [12]. Yet, if ever there was a time to reconsider the way we assess vaccines, it would be now, while we are facing the broad implications of a pandemic and shaping the post-COVID world.

To contribute a forward-looking perspective on what the implications may be of using a broader assessment framework, this study will answer the following questions: 1) How could a framework for broader value assessment of vaccines look like? 2) To what extent would including broader value elements in HTA be relevant for the vaccines that are coming through the pipeline in the near future? 3) To what extent do the current HTA frameworks allow to capture those broader values and where are the critical gaps? The study focuses on the UK, which has one of the world-leading immunisation programs. By identifying the gap between the broader value that might be expected from future vaccines to the UK and the readiness of the UK’s HTA bodies to capture it, the results aim to inform future methodological and policy discussions on the valuation of vaccination.

## 2 Material and methods

### 2.1 Overview

We used a multiple-methods approach including 1) a literature review to identify existing value frameworks for vaccines; 2) a review of the vaccine pipeline relevant to the UK; 3) a qualitative analysis of all value elements on which the vaccine would potentially have an incremental effect, and 4) a gap analysis comparing the results of step 3 to the value elements considered in the relevant HTA frameworks in the UK.

We sought feedback on the results of each step via interviews with eight experts: five interviewees had an industry background, two interviewees were experts in value assessment of vaccines and one interviewee was a representative of the UK’s Joint Committee on Vaccine and Immunisation (JCVI).

### 2.2 Literature review

We conducted a targeted literature search in quarters 3 and 4 of 2019 using the “snowballing” approach to identify peer-reviewed literature describing broader value elements or value frameworks for HTA of vaccines. Snowballing is an effective strategy for conducting a systematic yet targeted search [13]. It is based on selecting a relatively recent and authoritative paper as the starting point and identifying any articles that are cited in or have cited that paper. To this end, the conference report of Gessner et al. [11] was selected as the starting paper for the literature search as it describes the work and recommendations of a panel of 30 international experts on estimating the full public health value of vaccination. It references papers previously published on this topic and is referenced in authoritative journals like Vaccine. In addition, we searched Google Scholar to find other papers, using various terms such as: ‘vaccines economic value’, ‘vaccines economic assessment’, ‘vaccines health technology assessment’, and ‘vaccines HTA criteria’. From the papers found we extracted the value elements mentioned and their definitions. Given the geographical scope of this study, we focused on broader value elements that are most relevant in high-income settings like the UK. We organised these elements in a framework that underpins the next steps of this study.

### 2.3 Pipeline review

**Fig. 1:**
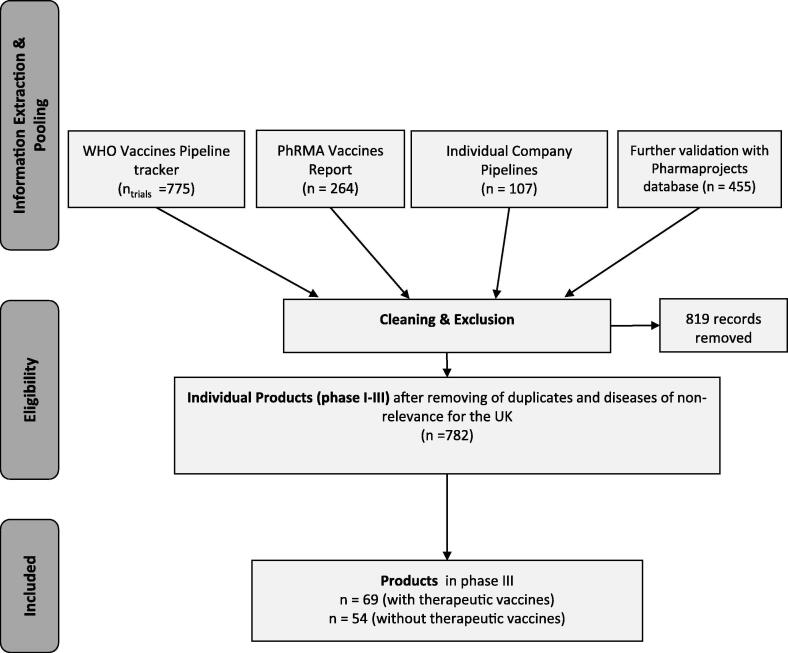
Flow chart of vaccines pipeline review.

The pipeline review was performed in quarters 3 and 4 of 2019 and based on three key sources of information including the publicly available vaccines pipeline trackers from the WHO^1^ and the Pharmaceutical Research and Manufacturers of America (PhRMA)^2^, and the commercial products pipeline database ‘Pharmaprojects’^3^. Vaccines in Pharmaprojects were identified using the following filters for therapeutic classes: prophylactic vaccine, anti-infective; therapeutic vaccine, anticancer, vaccine; recombinant vaccine. Duplicates between all sources were removed based on the product name, sponsor, and phase. We resolved instances of similar but non-identical names, and discrepancies in the reported development phase across datasets, using additional sources, notably company websites and clinicaltrials.gov. We excluded vaccines targeting diseases that currently have very low prevalence and incidence in the UK, including Dengue Fever, Ebola, Lassa Fever, Nipah Virus, MERS, Malaria, Zika, West Nile Virus, Plague / Yersinia Infections, although we are aware of the pandemic risk. We also excluded all vaccines that were not in Phase I, II or III development at the time of review. A flow chart of the review process is shown in Fig. 1.

The indications of the eligible vaccines were manually matched to the ICD-11 classification of the targeted disease. Due to different market access and HTA pathways, preventative and therapeutic vaccines in the pipeline were considered separately in the analyses. We distinguished preventative from therapeutic vaccines, by checking the product name, indication and, where available, information from clinicaltrials.gov using the keyword ‘prevention’ and ‘preventative’ or ‘therapy’, ‘treatment’, ‘therapeutic’. Unless specified otherwise, we assumed that every vaccine targeting neoplasms was a therapeutic vaccine. In any other case of doubt, the vaccine was classified as preventative.

We verified and complemented the final data extraction with input from representatives of eight vaccine manufacturers that are either large contributors to the global vaccine supply^4^ or major suppliers to the UK vaccination program.

### 2.4 Qualitative assessment of value elements relevant to pipeline vaccines

The qualitative assessment of a selection of pipeline vaccines was done by assessing on which value elements the selected vaccines would potentially generate value, and compared those value elements against the novel broader value framework. This approach was based on so-called ‘early HTA’ methodology [14], which aims to inform industry and other stakeholders about the potential value of new medical products in development. For this part of the analysis, we selected a sample of ten vaccines that were in phase III development. By focussing on phase III developments, we aimed to increase the significance of the analysis because these vaccines have a larger chance to successfully move to market and undergo HTA in the next couple of years. Also, while a set of 10 vaccines will not be fully representative for the entire pipeline, we strived to include a diverse set of vaccines by considering the characteristics of the vaccine-preventable disease (e.g. severity, prevalence and incidence, burden of disease), the target population (e.g. demographic, size) and whether it was a preventative or therapeutic vaccine.

As the impact of these ten phase III vaccines on the various value elements was unknown at the time of assessment, we searched for published data on the direct and indirect health and economic consequences of the specific diseases that the vaccines aim to prevent, to establish for which value elements there would be potential ‘headroom’ [15] for improvement. The assessment was binary (likely yes/ likely no) and does reflect the potential magnitude of the effect. We also sought feedback from the eight experts to face validate the assessment. We refer to the Appendix for a more detailed description of the value assessment for specific elements.

### 2.5 Gap analysis

The gap analysis involved reviewing HTA and cost-effectiveness methodology guidelines [16], [17], [18], [19] and previously published reviews [20] to list the value elements considered as part of the economic evaluation model or as additional criteria in the appraisals conducted by the JCVI, in charge of assessing preventative vaccines, and by the National Institute for Clinical Excellence (NICE), the organisation assessing therapeutic vaccines. We compared this list to the value elements on which the pipeline vaccines would have a potential impact, to identify the gaps. This provides insight into which value aspects might potentially be overlooked when these vaccines will be appraised using the current guidelines.

## 3 Results

### 3.1 Value frameworks for vaccines

The literature review identified four key publications reporting a description of value elements or a framework for assessing the full value of vaccines from a societal perspective [9], [21], [22], [23]. These frameworks differentiate between ‘narrow’ and ‘broad’ value elements of vaccines. Elements labelled as ‘narrow’, focus on (short-term) effects for vaccinated individuals and are considered in most vaccines HTAs. Elements labelled as ‘broad’ include downstream effects and externalities [21] and are not typically considered in most vaccine appraisals.

Building on previously published vaccines frameworks [9], [21], [22], [23], we distinguish four categories of value: (1) health effects, concerning the impact of vaccines on the health of vaccinated individuals and their informal caregivers, (2) productivity-related effects, concerning the impact of vaccines on the productivity of vaccinated individuals and their informal caregivers, (3) health system and community health impact, namely the impact of vaccines on the health of the unvaccinated population, and (4) health system economic effects. Each category includes multiple value elements that have previously been defined in the literature.

This study’s focus on higher-income countries led us to exclude value elements from previous frameworks. Examples of those elements are outcome-related productivity gains (i.e., changes to household fertility and consumption choices) and household security (i.e., avoidance of financial instability due to catastrophic health expenditure) which were considered more relevant to middle- and lower-income settings^5^. Table 1 shows the framework that was used for the remainder of this study.

**Table 1:** Value framework for vaccines.

**Categories**	**Value Elements**	**Definition**
Health effects	Impact on QoL of vaccinated individuals	Impact on patients’ physical, mental, emotional, and social functioning. It is hypothesised that the QoL will be affected by ‘peace of mind’ or ‘utility in anticipation’ benefits, occurring when a reduction in the fear of severe illness and associated disruptions to normal daily life has beneficial effects on the QoL of vaccinated individuals [24], [25].
	Impact on caregivers QoL	Impact on caregivers’ physical, mental, emotional, and social functioning. ‘Peace of mind’ and ‘utility in anticipation’ benefits are also relevant to caregivers [24]. In particular, parents of young children are expected to gain utility from the moment of vaccination until the time when the illness would have occurred, knowing that their children are protected against vaccine-preventable diseases [1].
	Impact on length of life of vaccinated individuals	Impact on length of life.
Productivity related impact	Impact on productivity of vaccinated individuals	Impact on work productivity due to sickness or death of the patient. Productivity losses may result from the impact on lost days of work and on the level of productivity, both for getting vaccinated and for disease avoided. In the case of the latter, it has been argued that vaccines can benefit from ‘outcome-related productivity’ by providing protection from diseases that can affect individuals’ ability to achieve/ maintain full cognitive potential, higher educational levels, and ultimately work productively during their lifetime [21].
	Impact on caregivers’ productivity	Impact on caregivers’ work productivity due to time spent caring for a sick individual.
Health system and community health impact	Burden of disease	The aggregate impact of disease in terms of total morbidity, as measured by disability-adjusted life years (DALYs) or quality adjusted life years (QALYs) lost. Consideration of burden of disease may also partly reflect societal preferences for equity. Where society places more value on the health gains accrued to worse off population groups, an efficiency-equity trade-off may improve the allocation of resources [26].
	Transmission value	Impact on disease transmission patterns and associated morbidity. Vaccines for infectious diseases can have an impact on population-wide epidemiological outcomes by providing herd immunity to unvaccinated individuals [21], [27].
	Prevention of antimicrobial resistance (AMR)	Impact on the rate of development and transmission of resistant infections. Vaccines targeting resistant bacterial infections can reduce the transmission and growth of AMR. Vaccines may also reduce the rate of prescription of antibiotics, thus slowing down the development of AMR [21], [27].
	Enablement value	Impact on the cost-effectiveness of other non-vaccine interventions. It has been argued that vaccines should not be evaluated in isolation because they enhance the effectiveness of other non-vaccine interventions [27]. For example, vaccinating patients with HBV or HCV viral load *and* concurrent cancer, may preserve the option to treat with a (higher intensity) chemotherapy, which would otherwise be a risk as chemotherapy might reactivate the virus [28].
Health system economic impact	Cost off-sets to the health system	Impact on medical costs borne by the health system, from a reduction in the number of medical consultations, treatment, screening, and hospitalisations. Compared to other health-related interventions, vaccines may also generate savings to the health system through a spending reduction on measures to prevent and control infection outbreaks [27], and, potentially, of fewer unnecessary clinic visits [22] due to ‘peace of mind’ benefits of vaccination [29], although evidence of the latter is currently unavailable.

### 3.2 Pipeline review

The pipeline review identified 782 products currently in phase I, II or III that are relevant to the UK. Of those, 478 (61%) were classified as preventative vaccines and 304 (39%) as therapeutic.

**Fig. 2:**
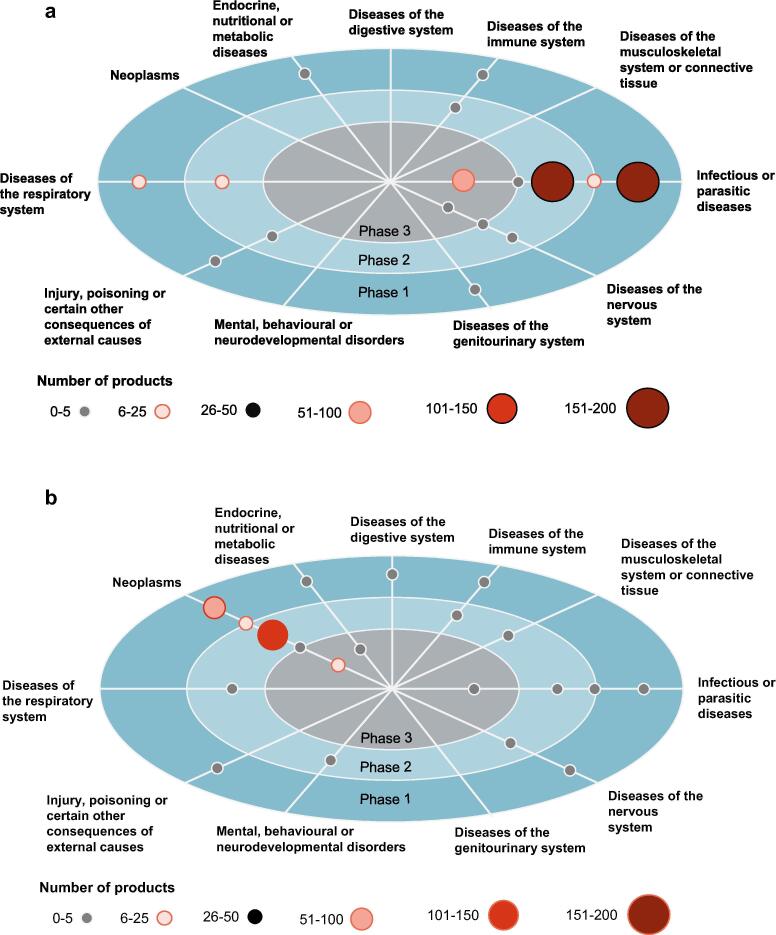
Results of the pipeline update: preventative (Panel A) and therapeutic (Panel B) vaccines in phase I, II or phase III development, by ICD-11 classified target disease.

For both types of vaccines, research and development (R&D) are heavily concentrated: ~90% of preventative vaccines in development target infectious or parasitic diseases (Fig. 2A) while 85% of therapeutic vaccines in the pipeline target neoplasms (Fig. 2B).

Among preventative vaccines, R&D activity centres on infectious or parasitic diseases (including influenza, pneumococcal disease and pentavalent vaccines for diphtheria, haemophilus influenzae type b, pertussis, polio and tetanus). This is especially visible in phase III, where this ICD-11 class captures 53 out of 54 ongoing developments. This is followed by products targeting diseases of the respiratory system (including meningococcal disease and tuberculosis) of which with 38 are mainly concentrated in phase I and II.

For therapeutic vaccines, most R&D activity targets neoplasms and is concentrated in phase II. Phase III includes vaccines that target prostate cancer (n = 2), liver cancer (n = 2) or multiple cancers (n = 3). There are also a few (0–5) developments in other ICD 11 classes, except for the genitourinary system.

### 3.3 Qualitative assessment of value elements relevant to pipeline vaccines

The results of the value assessment for the selected 10 vaccines in phase III are shown in Table 2. White cells indicate the value elements on which the vaccine might potentially have an incremental effect; grey cells indicate value elements that are not likely affected by the vaccine. In the case of burden of disease, a white cell indicates that the vaccine targets a disease which is in the top two deciles of the diseases contributing to the total UK disease burden; orange cells indicate it not.

**Table 2:** Value assessment and gap analysis of 10 selected vaccines.

Notes: White coloured cells: Potentially relevant value element (in case of ‘burden of disease’, the vaccines targets a disease that is in the top two decile of diseases contributing to the total UK disease burden); Grey coloured cells: Potentially irrelevant value element; (in case of ‘burden of disease’, the vaccine does not target a disease that is in the top two decile of diseases contributing to the total UK disease burden); **✓**Likely to be considered by JCVI/NICE; **X** Not likely to be considered by JCVI/NICE ; Cells with bolded black borders indicate value elements that are potentially relevant but unlikely to be considered by JCVI/NICE.

Among the health effects included in our framework, quality and length of life of vaccinated individuals were judged relevant to 10 and eight vaccines respectively, and QoL of caregivers to seven vaccines. Over half of the selected vaccines (n = 6) are expected to show community and health system effects relating to burden of disease, transmission and enablement value. Transmission value, in particular, is relevant to six out of the seven preventative vaccines in the sample. Prevention of AMR is likely to be relevant to four vaccines in the sample. The productivity impact of vaccinated individuals and caregivers were considered relevant to six and eight vaccines respectively, and cost-offsets to the health system to all 10 vaccines in the sample.

Below we provide the details of our assessment for one preventative (Escherichia Coli) and one therapeutic vaccine (Breast cancer). For similar descriptions of each vaccine assessment, we refer to the Appendix.

#### 3.3.1 Escherichia Coli (E. Coli)

Health effects: E. Coli infections can affect patients’ long-term QoL. In particular, patients’ mental health may be worse than the general population’s for six months [30] to one year [31] after recovering from the infection. E. Coli infections may also be responsible for the development of chronic conditions, e.g. chronic fatigue [31]. Most E. Coli infections improve in five to seven days, but more severe ones affecting children, elderly and immunocompromised individuals can be life-threatening [32]. Hence, a vaccine against E. Coli can *a priori* be expected to potentially impact QoL and length of life of patients. The length of E Coli infections episodes is considered short enough to exclude a significant impact on the QoL of informal caregivers.

Health system and community health impact: E. Coli is generally community-transmitted through consumption of contaminated foods [32]. In England, the incidence of E. Coli bacteraemia has increased, and about 60% of these cases have had a hospital-onset or have manifested in hospital-discharged patients with a history of healthcare interventions, such as urinary catheterization or antibiotic therapy within the previous four weeks [33], [34]. Consequently, an E. Coli vaccine is expected to deliver transmission value benefits.

While E. Coli is one cause of diarrhoeal disease and urinary tract infections, these conditions have a limited burden compared to other diseases contributing to the total UK disease burden (0.29% and 0.36% of total DALYs lost, respectively [35]). However, the bacterium is responsible for 84% of the total burden of bloodstream antibiotic-resistant infections in England [36], making prevention of AMR a relevant effect. Resistant E. Coli infections, for which antibiotic prophylaxis may not be effective, can prevent surgeries in immunocompromised patients. Hence, a vaccine against E. Coli is also expected to deliver gains in enablement value.

Health system economic impact: E. Coli infections result in physician visits, emergency department visits and hospitalisation. The estimated additional length of stay associated with E.Coli infections, relative to non-infections, is about four days. The estimated hospital costs to E. Coli bacteraemia in England is ~£14 million per year [37]. A vaccine against E.Coli is therefore expected to generate significant cost-offsets to the health system.

Productivity related impact: A study of the economic cost of E. Coli estimated that the number of days missed days from work ranges from 0.25 days, for patients that do not see a physician, to 7.13 days, for hospitalised patients [38]. Therefore, a vaccine may help to prevent productivity losses of infected individuals during the sickness period. The productivity of informal caregivers is assumed relatively unaffected.

#### 3.3.2 Breast cancer

Health effects: Breast cancer affects patients’ QoL in both short- and long-term. At diagnosis, patients may suffer mental distress, and during chemotherapy, they may experience fatigue and pain [39]. In the post-treatment stages, patients’ mental health may be affected by fear of recurrence, while physical QoL will generally be similar to the general population level, except for potential lymphedema and/or feeling of numbness to the arm [39]. Breast cancer caregivers’ may also be affected by mental distress and depression, conditions which are in turn correlated with lower QoL [40]. Five-year survival rates vary from 99% for diagnosis at stage one to 15% for diagnosis at stage four [41].

A therapeutic vaccine against breast cancer is thus expected to deliver value with respect to all health effects included in our framework.

Health system and community health impact: Breast cancer is the most common cancer in the UK, accounting for 15% of all new cancer cases in 2016. It ranks third among the diseases responsible for the largest number of years of life lost in women and among the top-10 diseases for the number of DALYs lost in women in the UK [42]. Some treatments for breast cancer can prevent or interfere with other treatments, particularly in severely immunosuppressed patients [28]. Overall, a vaccine against breast cancer is expected to reduce the burden of disease and have a relevant enablement value. The value of preventing disease transmission and AMR will likely not be relevant because of the non-communicable nature of breast cancer, and the use of antibiotics is limited to surgery prophylaxis.

Health system economic impact: Between 2006 and 2010, in England, breast cancer reportedly costs the health system £371 million in patients aged < 64 years [43]. Health system’s cost-offsets from a vaccine are expected to be significant.

Productivity related impact: Women with breast cancer or undergoing treatment report substantial impact on both work absenteeism and presenteeism [44]. Men whose partners are affected by breast cancer are also significantly more likely to stop working during treatment [45]. Productivity losses appear to dissipate for informal caregivers in the long term [45], but they can persist for survivors, even three years after treatment [46]. Thus, a vaccine against breast cancer is expected to enhance productivity gains for patients and informal caregivers.

### 3.4 Gap analysis

Table 2 also shows which value elements included in this study’s framework are likely to be considered in the JCVI appraisal of preventative vaccines and in the NICE appraisal of therapeutic ones. Cells marked with a dark blue triangle (**▴**), indicate that the value element is considered ‘likely to be included in the appraisal’; a black cross (**X**) indicated that the value element is ‘not likely to be included in the appraisal’. Potential areas of discrepancy between the relevant elements of value of vaccines and those that do not enter the vaccines appraisal are then apparent by light grey cells marked with a black cross.

Table 2 shows that all value elements related to health effects and health system economic impact are recognised by JCVI and NICE.

Areas of discrepancy appear to AMR prevention and enablement value, which neither JCVI nor NICE currently seem to recognise. The former is relevant to four of the seven preventative vaccines, but not to therapeutic vaccines. Enablement value is an area of value for three preventative and three therapeutic vaccines.

Productivity related effects of vaccinated individuals and caregivers are not considered by JCVI or NICE. This creates areas of discrepancy among both preventative and therapeutic vaccines.

## 4 Discussion

The current vaccines pipeline shows significant activity: 23 preventative and seven therapeutic vaccines that are currently in phase III may be launched in the UK within the next five years. While an appropriate assessment is crucial to assure sufficient uptake and reimbursement, our results show that some broader value aspects potentially generated by a selection of these future vaccines are not likely to be considered in the current NICE or JCVI appraisals. It should be noted that other health technologies may also accrue broader value; however, the broader value component of vaccines is unusually large compared to other many health technologies. The identified gaps essentially represent a systematic undervaluation of vaccination, which has detrimental effects on long-term population health. Undervaluation may lead to underinvestment and underutilisation, which may leave our society vulnerable to diseases that could potentially be prevented through vaccination. This scenario seems especially grim given the added health system pressure from the ongoing COVID-19 pandemic.

It must be noted, however, that JCVI and NICE may consider, on a case-by-case basis, some of the broader value elements. For example, if a significant health benefit or cost-saving is known to exist but is not included in the modelling, the JCVI is willing to use their judgement to adjust the estimates of the incremental cost-effectiveness ratio [22]. Also, a previously published review [47] of NICE assessments shows that carer or family member utilities were included or commented on in five of 58 appraisals completed in 2018. While encouraging examples, current practice does not assure a consistent and transparent inclusion of broader value elements and may distort value comparisons between health-related interventions. Furthermore, in the past NICE has rejected the use of other methodological alternatives, such as multi-criteria decision analysis, that would allow to consider broader value elements alongside the results of an economic evaluation model. There are, however, other ways to overcoming the identified gaps. Methodological advancements such as dynamic disease modelling already allow to formally incorporate transmission value on a regular basis. Others would require a more systemic change in existing approaches. We summarise those below and refer to a working paper^6^ for further detailed recommendations.

First, for preventative vaccines, while not currently consistently included, the JCVI acknowledges the importance of considering the potential of vaccines to prevent AMR and/or to improve the cost-effectiveness of other non-vaccine interventions (enablement value). Regarding AMR, substantial cost-savings might be achieved given their large associated cost-burden. However, a better understanding of the patterns of AMR development and models is needed to quantify the impact of relevant vaccination programmes. The JCVI has already issued a recommendation for generating such evidence [18]. For enablement value, an explicit and consistent inclusion would allow capturing the value of vaccination that is generated by enabling other cost-effective interventions. This requires a clear definition of what this value element includes, and methodologies to measure it accurately. A possible solution may be the joint evaluation of the vaccines with the non-vaccine interventions that will be ‘enabled’ [27].

Second, possibilities to capture this value by broadening the perspective would be allowed under the NICE public health guidance [48]. Here, evaluation methods such as cost-benefit analysis can be applied, which have been found to be more adequate for the evaluation of vaccines [49] as they allow for including relevant broader elements. Alternatively, the existing methods applied by NICE and the JCVI could be complemented with fiscal health analyses [50] which would include the value of vaccination from a treasury perspective. If justified, the treasury could then reallocate sufficient funds to the health system to ensure the affordability of evaluated vaccination program.

Third, the value dimensions of the broader value framework may not be of equal relevance to the different stakeholders. Those with responsibility for health planning, budget development, and management of community-based programmes may be more interested in the prevention of AMR, whereas for example the treasury, having with broader financial objectives, may place greater value on vaccines’ productivity-related value. Therefore, allocative efficiency may be improved by considering who gains and who pays for vaccination. Expanding employer-funded vaccination programs to other vaccine-preventable diseases than flu, and potentially open these up to family members if there are substantial carer productivity costs associated with the disease, is one example.

This research has limitations. The updated pipeline may exhibit some redundancy if, despite multiple automatic and manual checks, some duplicate products were not detected.

The mapping of vaccines to ICD 11 classes skews towards “Infectious diseases” as this captures every disease that spreads, directly or indirectly, from one person to another.

The methods used to perform the value assessment are prone to selection and publication bias. We based the qualitative assessment on published data where possible, yet the data availability and quality tended to be stronger for the direct health effects than for the ‘broader’ value elements; a consequence in part of the status quo. We aimed to mitigate this by complementing the data available with expert judgements from representatives of public health authorities and the pharmaceutical industry. Also, in the absence of clear target product profiles, this part of the assessment involved value judgements as to whether the vaccine could *potentially* generate added value, considering the counterfactual of current care without that vaccine. This does not imply that the vaccine will indeed deliver that value, which remains to be demonstrated in further research.

## 5 Conclusion

To our knowledge, this is the first time that the potential broader value of vaccination has been pro-actively assessed for pipeline vaccinations. Our findings show that the existing evaluation frameworks are likely to systematically undervalue the vaccines coming to market in the UK. This is particularly the case where their impact on AMR, on other health interventions, and on the productivity of the workforce is of concern. Recommendations to overcome this include an explicit and more consistent inclusion of, and data collection on, the impact of vaccines on AMR and other health interventions by JCVI and NICE, and consideration of a societal perspective and the fiscal impact of vaccines to societies.

## Declaration of Competing Interest

The authors declare the following financial interests/personal relationships which may be considered as potential competing interests: All authors are employees of The Office of Health Economics (London, UK), a registered charity and Independent Research Organisation which receives funding from a variety of private and public sector sources. The underlying research was commissioned and funded by the Association of the British Pharmaceutical Society (ABPI).

## Footnotes

1 WHO vaccine pipeline tracker. http://www.who.int/immunization/research/vaccine_pipeline_tracker_spreadsheet/en/ (accessed October 16, 2019).

2 PhRMA. Medicines in Development: Vaccines. 2017.

3 Pharma Intelligence. Pharmaprojects n.d. http://pharmaintelligence.informa.com/products-and-services/data-and-analysis/pharmaprojects (accessed October 16, 2019).

4 Based on a report published by the Access to Medicine Foundation “Access to Vaccines Index (2017)” Available at https://accesstomedicinefoundation.org/access-to-vaccines-index/about-the-index.

5 We note that this part of the study had been performed before the COVID-19 pandemic

6 See Brassel S, Neri M, O’Neill P, Steuten L. Realising the Value of Vaccines in the UK. London: Office of Health Economics; 2020. Available at: https://www.ohe.org/publications/realising-broader-value-vaccines-uk
