# Supplementary material for: Realising the broader value of vaccines in the UK
Source: Vaccine X. 2021 Apr 6;8:100096. doi: 10.1016/j.jvacx.2021.100096 (PMC8099625; doi:10.1016/j.jvacx.2021.100096)
Supplement: Supplementary data 1 [file mmc1.docx]

# Appendix: Value assessment supporting material

**Table B1. Value assessment results (Part 1 of 2)**

| **DISEASE** | **POPULATION,**  **VACCINE TYPE** | **VALUE ELEMENTS – Part 1 of 2** | | | | | |
| --- | --- | --- | --- | --- | --- | --- | --- |
|  |  | **Impact on the QoL of vaccinated individuals** | **Impact on QoL of caregivers** | **Impact on length of vaccinated individuals** | **Impact on productivity of vaccinated individuals** | **Impact on productivity of caregivers** |  |
| **HIV** | Adults, Preventative | - HIV is nowadays a chronic and manageable disease. HIV may however cause anxiety and depression more than other chronic diseases^1^ | - HIV caregivers may report poorer psychological health^2^ | - If untreated, HIV is a deadly condition but - with modern treatments, survival rates of people with HIV have improved substantially in the past two decades^3^ | - Productivity of HIV patients who work is overall similar to that of healthy people^4^ - However, HIV is responsible for lower employment rates and premature mortality^4^ | - Caregiver productivity is likely to decrease in the late stages of the disease when the health of the patient is deteriorating^5^ |  |
| **Influenza** | Children, Adults, Preventative | - Influenza symptoms can be debilitating during the illness period, but patients are typically able to regain full health after recovery^6^ | - Influenza is a curable disease and the episode length is moderate enough to exclude a significant impact on QoL of caregivers^5^ | - In developed countries, influenza can cause severe illness or death people aged 65 or older^7^ | - Productivity losses resulting from missed work days can be frequent but are limited to a few days per episode | - Children are likely to require someone else to take care of them, normally adults in working age^8^ |  |
| **Alzheimer** | Adults, Preventative | - Progressive deterioration of brain functions. In late stages patients become completely dependent on caregivers^9^ | - Physical and mental distress of caregivers increase in the later stages of disease, when patients become completely dependent on caregivers^10^ | - Impact on length of life may depending on the age of onset, stage of the disease when diagnosed^9^ | - Prevalence of disease risk is highest among the retired, non-working population^9^ | - Early disease stage requires minor carer support, while patients become completely dependent on caregivers in later disease stages^9^ |  |
| **Respiratory syncytial virus (RSV) infection** | Adults, Senior, Preventative | - In healthy individuals, RSV symptoms are mild and typically mimic a common cold. However, RSV can also cause severe infections in the elderly^11^ | - RSV associated diseases are curable, and length of disease episode is moderate enough to exclude a significant impact on QoL of informal caregivers^5^ | - Among the elderly, RSV infection has a significantly higher risk of death compared to seasonal influenza^11^ | - The most severe RSV infections are among the retired elderly^11^ | - Productivity of caregivers may be impacted because the most severe RSV infections requiring care occur among the elderly^5^ |  |
| **Escherichia coli infection** | Adults, Preventative | - E. coli infection symptoms include stomach cramps, diarrhoea and vomiting. Most infections get better in 5-7 days, but more severe ones can be life-threatening^12^ | - The length of E Coli infections episodes is typically moderate enough to exclude a significant impact on QoL of informal caregivers^5^ | - E. Coli may lead to life-threatening complications in children, the elderly and immunocompromised individuals^12^ | - Severe episodes of E. Coli may require hospitalisation periods that require time off work, or cause chronic disabilities^12^ | - Treatment of severe cases takes place in hospital, hence impact on caregivers' productivity is unlikely to be significant^5^ |  |
| **Meningococcal infection** | Children, Preventative | - The majority of patients recovers completely but aggressive forms of meningitis can leave survivors with long term disabilities^13^ | - Parents suffer mental distress when their children become and in the long-term when their children survive with impairing sequelae^13^ | - Meningitis is a potentially life-threatening disease^13^ | - Productivity losses may be due to premature death and long term disabilities^14^ | - Caregivers of children with meningitis will incur productivity losses during the hospitalisation period and subsequent check-ups^15^ |  |
| **Varicella zoster virus infection** | Children, Preventative | - Chickenpox in children is responsible for fever and low energy levels^16^ | - Chickenpox episodes are of moderate length, thus not affecting significantly caregivers QoL of caregivers expected^5^ - Caregiving burden due to herpes zoster in adults is limited^5^ | - Risk of death is higher among immunocompromised children and adults, but low otherwise^17^ | - Varicella impact on quality of life is unlikely to have implications for the long-term productivity of patients^5^ | - Parents may miss a few days of work to attend a sick child with chickenpox^5^ |  |
| **Breast cancer** | Adult, Therapeutic | - At diagnosis, patients may suffer mental distress, and during chemotherapy they may experience fatigue and pain^19^ - In the post-treatment stages, patients’ mental health may be affected by fear of recurrence^19^ | - Breast cancer caregivers’ may also be affected by mental distress and depression^20^ | - Five-year survival rates vary to a significant degree: from 99% for diagnosis at stage one disease to 15% for diagnosis at stage four disease^21^ | - Breast cancer can lead to premature mortality, work absenteeism and premature retirement^22^ | - Caregiver involvement in household activities may increase as a result of the patient's disease, thus decreasing caregiver work productivity^23^ |  |
| **Diabetes,  Type 2** | Senior, Therapeutic | - Diabetes type 2 is treated with regular medication but may lead to severe complications, such as renal failure, coronary arterial disease, blindness^24^ | - Caregivers of type 2 diabetes may be more likely to have depression^25^ | - Diabetes type 2 complications may impact patients’ length of lie, but a timely diagnosis and effective management patients can achieve normal life expectancy levels^26^ | - Evidence of absenteeism, disability and premature retirement of patients approaching the retirement age^27^ | - Impact on productivity of caregivers likely to increase with the risk of complications and patient's age^5^ |  |
| **Prostate cancer** | Adult, Therapeutic | - Prostate-cancer and its treatment can impact quality-of-life domains related to urinary, sexual, bowel, and hormonal function^28^ | - Prostate cancer and its treatment can cause spousal distress and dissatisfaction with the treatment outcome^28^ | - High rates of 5-year survival rate in the UK, but higher mortality rates with more aggressive or later diagnosed cancers^29^ | - Prostate cancer incidence higher in non-working patient population, aged 75-79 years^29^ | - Caregiver involvement in household activities may increase as a result of the patient's disease, thus decreasing caregiver work productivity^23^ |  |

Notes: 1 (Engelhard et al., 2018); ^2^(Marc et al., 2011); ^3^(Trickey et al., 2017); 4(Verbooy et al., 2018); 5Expert judgement; ^6^(Hollmann et al., 2013); ^7^(WHO, 2018b); ^8^(Fragaszy et al., 2018); ^9^(Luengo-Fernandez, Leal and Gray, 2010); ^10^(Rees, O’boyle and MacDonagh, 2001); ^11^(CDC, 2018); ^12^(WHO, 2018a); ^13^(Olbrich et al., 2018); ^14^(WHO, n.d.); ^15^(Chen et al., 2019); ^16^(Gershon et al., 2015); ^17^(CDC, n.d.); ^18^(Bilcke et al., 2012); ^19^(Paraskevi, 2012); ^20^(Gorji et al., 2012); ^21^(Cancer Research UK, 2017); ^22^(Frederix et al., 2013); ^23^(Kamal et al., 2017); ^25^(Anaforoğlu et al., 2012); ^26^(Diabetes.co.uk, 2019)^; 27^(Breton et al., 2012); ^28^(Sanda et al., 2008); ^29^(Prostate Cancer Foundation, n.d.)

Key: White coloured cells: Potentially relevant value elements (in case of ‘burden of disease’ this means that the vaccine targets a disease that is in the top two decile of diseases contributing to the total UK disease burden); Greu coloured cells: Potentially irrelevant (in case of ‘burden of disease’ this means that the vaccine does not target a disease that is in the top two decile of diseases contributing to the total UK disease burden).

**Table B2: Value assessment results (Part 2 of 2)**

| **DISEASE** | **POPULATION,**  **VACCINE TYPE** | **VALUE ELEMENTS (Part 2 of 2)** | | | | |
| --- | --- | --- | --- | --- | --- | --- |
|  |  | **Burden of disease*** | **Enablement Value** | **Transmission value** | **Prevent AMR development** | **Cost-offsets to healthcare system** |
| **HIV** | Adults, Preventative | - Sum of burden of HIV disease and HIV complications amounts to 0.1% of total DALYs lost in the UK^1^ | - Untreated HIV will damage the immune system thus compromising the treatment of other diseases (e.g. chemotherapy for cancer), but currently it is possible to treat HIV with antivirals^2^ | - HIV is transmitted via certain body fluids from infected individuals^3^ | - HIV is a viral disease, not associated to antibiotic use^2^ | - Risk of disease complications related to impairment of immune system may require hospitalisation^2^ |
| **Influenza** | Children, Adults, Preventative | - In 2009-2013, influenza was responsible for 29.8% of the total number of DALYs lost in Europe^4^ | - Influenza is unlikely to cause complications in children and adults that prevent the treatment of diseases associated to other comorbidities^2^ | - Transmission of influenza can be high in certain seasons of the year, typically winter^5^ | - Episodes of influenza are associated to inappropriate prescription of antibiotics^6^ | - Risk of hospitalisation is higher among weaker patient groups, but outpatient costs are also high^7^ |
| **Alzheimer** | Adults, Preventative | - Alzheimer ranks 5th among the diseases responsible for the highest percentage of DALYs lost in the UK (3.46%)^1^ | - Alzheimer is unlikely to compromise the immune system of patients to the point of preventing the treatment of other diseases^2^ | - Alzheimer is a non-communicable disease^2^ | - Alzheimer is a non-communicable disease, not associated to antibiotic use^2^ | - Need of medical care and social services increase in the late stages of the disease^8^ |
| **Respiratory syncytial virus (RSV) infection** | Adults, Senior, Preventative | - RSV is one of the leading causes of lower respiratory tract infections (bronchiolitis and pneumonia), which are among the top 5% diseases with the highest burden of disease in the UK (2.11%)^1^ | - Due to the lack of treatment for RSV, severe infections in the weaker population groups may prevent the treatment of disease associated to other comorbidities^2^ | - RSV infections are transmitted via coughs or sneezing from infected people ^9^ | - Even though the rates of bacterial infections complicating RSV are rare, antibiotics are often prescribed in children with viral RSV bronchiolitis^10^ | - Greatest source of use health resources due to RSV infection is from outpatient visits^11^ |
| **Escherichia coli infection** | Adults, Preventative | - E. Coli is one of the multiple causes of diarrheal diseases, which overall are 0.22% of the total DALYs lost in the UK^1^ | - Resistant E. Coli infections for which antibiotics do not work may prevent surgeries in patients with compromised immune systems^2^ | - E Coli is transmitted to humans primarily through consumption of contaminated foods^12^ | - E. Coli is listed in the 'critical' group of the WHO priority pathogens list^13^ | - E. Coli infections can result in physician visits, emergency department visits and hospitalisation^14^ |
| **Meningococcal infection** | Children, Preventative | - The sum of burden of meningococcal disease and related complications is about 0.08% of the total DALYs lost in the UK^1^ | - A meningococcal vaccine is required before starting treatment for haemolysis and thrombotic microangiopathy^24^ | - Meningitis can be spread by contact with infected people^15^ | - Many of the bacteria included in the list compiled by WHO can cause bacterial meningitis^16^ | - Short- and long-term medical cost of meningitis disabilities from severe cases are large^17^ |
| **Varicella zoster virus infection** | Children, Preventative | - Burden of varicella and herpes zoster is about 0.02% of total DALYs lost in the UK^1^ | - Varicella and herpes zoster are unlikely to prevent the treatment of other diseases in the affected patient^2^ | - The virus spread via close contact with infected people^18^ | - Varicella zoster infections are viral and not associated to antibiotic use^2^ | - Varicella can result in significant burden to the health system in terms of physician visits and hospitalisation^19^ |
| **Breast cancer** | Adult, Therapeutic | - Breast cancer ranks among the top-10 diseases for number of DALYs lost in women in the UK^20^ | - Systemic cancer treatment is likely to compromise the immune system of patients to the point of preventing the treatment of other diseases^2^ | - Breast cancer is a non-communicable disease^2^ | - Breast cancer is not associated to antibiotic use^2^ | - Medical costs of breast cancer increase with the disease stage at diagnosis^21^ |
| **Diabetes,  Type 2** | Senior, Therapeutic | - Diabetes is in the top 5% of diseases responsible for the largest DALYs loss in the UK (2.32%)^1^ | - Diabetes complications, particularly among the elderly, may prevent the treatment of other comorbidities^2^ | - Diabetes is a non-communicable disease^2^ | - Diabetes is not associated to antibiotic use^2^ | - Diabetes accounts for about 10 per cent of the NHS budget and 80 per cent of these costs are due to complications^22^ |
| **Prostate cancer** | Adult, Therapeutic | - Prostate cancer is in the top 10% of diseases responsible for the largest burden of disease in the UK (1.06%)^1^ | - Systemic cancer treatment is likely to compromise the immune system of patients to the point of preventing the treatment of other diseases^2^ | - Prostate cancer is a non-communicable disease^2^ | - Prostate cancer is not associated to antibiotic use^2^ | - Prostate cancer is associated with increased direct healthcare costs over the natural history of the disease. Costs are highest around cancer diagnosis and cancer death^23^ |

Notes: * To qualify the burden of individual diseases to the UK, we considered the total burden of disease in the UK. For each disease, we obtained data on DALYs lost in the UK from the latest issue of the Global Burden of Disease study (Institute for Health Metrics and Evaluation, 2017). The DALY combines the years of life lost (YLL) due to premature mortality and the years of life lost due to disability (YLD) for people living with a specific health condition or its consequences. First, we sorted the diseases in increasing order by their percentage contribution to the total DALYs lost in the UK. We then applied the ‘Pareto principle’ as our classification rule to determine whether the burden of disease is particularly relevant to the UK. The Pareto principle establishes that roughly 80% of the effects (in this case the total UK burden in DALYs) stems from the top 20% of the causes (in this case the diseases) (Pareto and Page, 1971). Therefore, it follows that the bottom 80% of diseases are individually responsible for minor burden, because collectively they only account for 20% of the total burden, in DALYs, in the UK. Based on this we highlighted the diseases appearing in the top 20% of them as ‘particularly relevant’ in the assessment.

^1^(Institute for Health Metrics and Evaluation, 2017); ^2^Expert judgement; ^3^(WHO, 2019); ^4^(Cassini et al., 2016); ^5^(WHO, 2018b); ^6^(Havers et al., 2018); ^7^(Federici et al., 2018); ^8^(Castro et al., 2010); ^9^(CDC, 2018); ^10^(Quintos-Alagheband et al., 2017); ^11^(Amand et al., 2018); ^12^(WHO, 2018a); ^13^(Tacconelli et al., 2017); ^14^(Frenzen et al., 2005); ^15^(NHS, 2019); ^16^(Meningitis Research Foundation, 2018); ^17^(Wright, Wordsworth and Glennie, 2013); ^18^(CDC, n.d.); ^19^(Brisson and Edmunds, 2003); ^20^(Steel et al., 2018); ^21^(Sun et al., 2018); ^22^(Diabetes UK, 2014); ^23^(Krahn et al., 2010),^24^ (NICE, n.d.)

Key: White coloured cells: Potentially relevant value elements (in case of ‘burden of disease’ this means that the vaccine targets a disease that is in the top two decile of diseases contributing to the total UK disease burden); Greu coloured cells: Potentially irrelevant (in case of ‘burden of disease’ this means that the vaccine does not target a disease that is in the top two decile of diseases contributing to the total UK disease burden).

**Sources**

Amand, C., Tong, S., Kieffer, A. and Kyaw, M.H., 2018. Healthcare resource use and economic burden attributable to respiratory syncytial virus in the United States: a claims database analysis. *BMC health services research*, 18(1), p.294.

Anaforoğlu, İ., Ramazanoğulları, İ., Algün, E. and Kutanis, R., 2012. Depression, anxiety and quality of life of family caregivers of patients with type 2 diabetes. *Medical Principles and Practice*, 21(4), pp.360–365.

Bilcke, J., OGUNJIMI, B., Marais, C., De Smet, F., Callens, M., Callaert, K., Van Kerschaver, E., Ramet, J., Van Damme, P. and Beutels, P., 2012. The health and economic burden of chickenpox and herpes zoster in Belgium. *Epidemiology & Infection*, 140(11), pp.2096–2109.

Breton, M.-C., Guenette, L., Amiche, M.A., Kayibanda, J.-F., Gregoire, J.-P. and Moisan, J., 2012. The Burden of Type 2 Diabetes on Work Productivity: A Systematic Review. *Canadian Journal of Diabetes*, 36(5), p.S71. 10.1016/j.jcjd.2012.07.451.

Brisson, M. and Edmunds, W., 2003. Varicella vaccination in England and Wales: cost-utility analysis. *Archives of disease in childhood*, 88(10), pp.862–869.

Cancer Research UK, 2017. *Breast Cancer. Survival*. [online] Available at: https://www.cancerresearchuk.org/about-cancer/breast-cancer/survival .

Cassini, A., Plachouras, D., Eckmanns, T., Sin, M.A., Blank, H.-P., Ducomble, T., Haller, S., Harder, T., Klingeberg, A. and Sixtensson, M., 2016. Burden of six healthcare-associated infections on European population health: estimating incidence-based disability-adjusted life years through a population prevalence-based modelling study. *PLoS medicine*, 13(10), p.e1002150.

Castro, D.M., Dillon, C., Machnicki, G. and Allegri, R.F., 2010. The economic cost of Alzheimer’s disease: Family or public-health burden? *Dementia & neuropsychologia*, 4(4), pp.262–267.

CDC, 2018. *Respiratory Syncytial Virus Infection (RSV)*. [online] Available at: https://www.cdc.gov/rsv/index.html .

CDC, n.d. *Varicella*. [online] Available at: https://www.cdc.gov/vaccines/pubs/pinkbook/varicella.html .

Chen, C., Cervero Liceras, F., Flasche, S., Sidharta, S., Yoong, J., Sundaram, N. and Jit, M., 2019. Effect and cost-effectiveness of pneumococcal conjugate vaccination: a global modelling analysis. *The Lancet Global Health*, 7(1), pp.e58–e67. 10.1016/S2214-109X(18)30422-4.

Diabetes UK, 2014. *The cost of diabetes*. [online] Available at: https://www.diabetes.org.uk/resources-s3/2017-11/diabetes%20uk%20cost%20of%20diabetes%20report.pdf .

Diabetes.co.uk, 2019. *Diabetes life expectancy*. [online] Available at: https://www.diabetes.co.uk/diabetes-life-expectancy.html .

Engelhard, E.A., Smit, C., Van Dijk, P.R., Kuijper, T.M., Wermeling, P.R., Weel, A.E., De Boer, M.R., Brinkman, K., Geerlings, S.E. and Nieuwkerk, P.T., 2018. Health-related quality of life of people with HIV: an assessment of patient related factors and comparison with other chronic diseases. *Aids*, 32(1), pp.103–112.

Federici, C., Cavazza, M., Costa, F. and Jommi, C., 2018. Health care costs of influenza-related episodes in high income countries: A systematic review. *PloS one*, 13(9), p.e0202787.

Fragaszy, E.B., Warren‐Gash, C., White, P.J., Zambon, M., Edmunds, W.J., Nguyen‐Van‐Tam, J.S., Hayward, A.C. and Flu Watch Group, 2018. Effects of seasonal and pandemic influenza on health‐related quality of life, work and school absence in England: Results from the Flu Watch cohort study. *Influenza and other respiratory viruses*, 12(1), pp.171–182.

Frederix, G.W., Quadri, N., Hövels, A.M., van de Wetering, F.T., Tamminga, H., Schellens, J.H. and Lloyd, A.J., 2013. Utility and work productivity data for economic evaluation of breast cancer therapies in the Netherlands and Sweden. *Clinical therapeutics*, 35(4), pp.e1–e7.

Frenzen, P.D., Drake, A., Angulo, F.J. and Emerging Infections Program FoodNet Working Group, 2005. Economic cost of illness due to Escherichia coli O157 infections in the United States. *Journal of food protection*, 68(12), pp.2623–2630.

Gershon, A.A., Breuer, J., Cohen, J.I., Cohrs, R.J., Gershon, M.D., Gilden, D., Grose, C., Hambleton, S., Kennedy, P.G. and Oxman, M.N., 2015. Varicella zoster virus infection. *Nature reviews Disease primers*, 1, p.15016.

Gorji, M.A.H., Bouzar, Z., Haghshenas, M., Kasaeeyan, A.A., Sadeghi, M.R. and Ardebil, M.D., 2012. Quality of life and depression in caregivers of patients with breast cancer. *BMC research notes*, 5(1), p.310.

Havers, F.P., Hicks, L.A., Chung, J.R., Gaglani, M., Murthy, K., Zimmerman, R.K., Jackson, L.A., Petrie, J.G., McLean, H.Q. and Nowalk, M.P., 2018. Outpatient antibiotic prescribing for acute respiratory infections during influenza seasons. *JAMA network open*, 1(2), pp.e180243–e180243.

Hollmann, M., Garin, O., Galante, M., Ferrer, M., Dominguez, A. and Alonso, J., 2013. Impact of influenza on health-related quality of life among confirmed (H1N1) 2009 patients. *PloS one*, 8(3), p.e60477.

Institute for Health Metrics and Evaluation, 2017. *Global Burden of Disease Study - United Kingdom*. [online] Available at: http://www.healthdata.org/united-kingdom .

Kamal, K.M., Covvey, J.R., Dashputre, A., Ghosh, S., Shah, S., Bhosle, M. and Zacker, C., 2017. A systematic review of the effect of cancer treatment on work productivity of patients and caregivers. *Journal of managed care & specialty pharmacy*, 23(2), pp.136–162.

Krahn, M.D., Zagorski, B., Laporte, A., Alibhai, S.M., Bremner, K.E., Tomlinson, G., Warde, P. and Naglie, G., 2010. Healthcare costs associated with prostate cancer: estimates from a population‐based study. *BJU international*, 105(3), pp.338–346.

Luengo-Fernandez, R., Leal, J. and Gray, A., 2010. Dementia 2010: The economic burden of dementia and associated research funding in the United Kingdom. *Cambridge: Alzheimer’s Research Trust*.

Marc, L.G., Zerden, M., Ferrando, S.J. and Testa, M.A., 2011. HIV+ caregivers and HIV+ non-caregivers: differences in sociodemographics, immune functioning, and quality-of-life. *AIDS care*, 23(7), pp.880–891.

Meningitis Research Foundation, 2018. *What happens when antibiotics don’t treat meningitis anymore?* [online] Available at: https://www.meningitis.org/blogs/meningitis-antibiotic-resistance .

NHS, 2019. *Meningitis - Overview*. [online] Available at: https://www.nhs.uk/conditions/meningitis/ .

NICE, n.d. *ECULIZUMAB*. [online] Available at: https://bnf.nice.org.uk/drug/eculizumab.html .

Olbrich, K.J., Müller, D., Schumacher, S., Beck, E., Meszaros, K. and Koerber, F., 2018. Systematic review of invasive meningococcal disease: sequelae and quality of life impact on patients and their caregivers. *Infectious diseases and therapy*, 7(4), pp.421–438.

Paraskevi, T., 2012. Quality of life outcomes in patients with breast cancer. *Oncology reviews*, 6(1).

Pareto, V. and Page, A.N., 1971. Translation of Manuale di economia politica. In: *Manual of political economy*. AM Kelley.

Prostate Cancer Foundation, n.d. *Incidence of Prostate Cancer*. [online] Available at: https://www.pcf.org/about-prostate-cancer/what-is-prostate-cancer/prostate-cancer-survival-rates/ .

Quintos-Alagheband, M.L., Noyola, E., Makvana, S., El-Chaar, G., Wang, S., Calixte, R. and Krilov, L.R., 2017. Reducing Antibiotic Use in Respiratory Syncytial Virus—A Quality Improvement Approach to Antimicrobial Stewardship. *Pediatric quality & safety*, 2(6).

Rees, J., O’boyle, C. and MacDonagh, R., 2001. Quality of life: impact of chronic illness on the partner. *Journal of the Royal Society of medicine*, 94(11), pp.563–566.

Sanda, M.G., Dunn, R.L., Michalski, J., Sandler, H.M., Northouse, L., Hembroff, L., Lin, X., Greenfield, T.K., Litwin, M.S. and Saigal, C.S., 2008. Quality of life and satisfaction with outcome among prostate-cancer survivors. *New England Journal of Medicine*, 358(12), pp.1250–1261.

Steel, N., Ford, J.A., Newton, J.N., Davis, A.C., Vos, T., Naghavi, M., Glenn, S., Hughes, A., Dalton, A.M. and Stockton, D., 2018. Changes in health in the countries of the UK and 150 English Local Authority areas 1990–2016: a systematic analysis for the Global Burden of Disease Study 2016. *The Lancet*, 392(10158), pp.1647–1661.

Sun, L., Legood, R., dos-Santos-Silva, I., Gaiha, S.M. and Sadique, Z., 2018. Global treatment costs of breast cancer by stage: A systematic review. *PloS one*, 13(11), p.e0207993.

Tacconelli, E., Magrini, N., Kahlmeter, G. and Singh, N., 2017. Global priority list of antibiotic-resistant bacteria to guide research, discovery, and development of new antibiotics. *World Health Organization*, 27.

Trickey, A., May, M.T., Vehreschild, J.-J., Obel, N., Gill, M.J., Crane, H.M., Boesecke, C., Patterson, S., Grabar, S. and Cazanave, C., 2017. Survival of HIV-positive patients starting antiretroviral therapy between 1996 and 2013: a collaborative analysis of cohort studies. *The Lancet HIV*, 4(8), pp.e349–e356.

Verbooy, K., Wagener, M., Kaddouri, M., Roelofs, P., Miedema, H., van Gorp, E., Brouwer, W. and van Exel, J., 2018. Are people living with HIV less productive at work? *AIDS care*, 30(10), pp.1265–1272.

WHO, 2018a. *E. Coli*. [online] Available at: https://www.who.int/news-room/fact-sheets/detail/e-coli .

WHO, 2018b. *Influenza (Seasonal)*. [online] Available at: https://www.who.int/news-room/fact-sheets/detail/influenza-(seasonal) .

WHO, 2019. *HIV/AIDS*. [online] Available at: https://www.who.int/news-room/fact-sheets/detail/hiv-aids .

WHO, n.d. *Global Health Observatory (GHO) data - Number of suspected meningitis cases and deaths reported*. [online] Available at: https://www.who.int/gho/epidemic_diseases/meningitis/suspected_cases_deaths_text/en/ .

Wright, C., Wordsworth, R. and Glennie, L., 2013. Counting the cost of meningococcal disease. *Pediatric Drugs*, 15(1), pp.49–58.
